# Porcine parvovirus triggers autophagy through the AMPK/Raptor/mTOR pathway to promote viral replication in porcine placental trophoblasts

**DOI:** 10.1186/s13567-022-01048-7

**Published:** 2022-05-03

**Authors:** Xiujuan Zhang, Peipei Ma, Ting Shao, Yingli Xiong, Qian Du, Songbiao Chen, Bichen Miao, Xuezhi Zhang, Xiaoya Wang, Yong Huang, Dewen Tong

**Affiliations:** College of Veterinary Medicine, Northwest A&F University, Yangling, 712100 China

**Keywords:** Porcine parvovirus, placental trophoblast cells, autophagy, signalling pathway

## Abstract

Autophagy has been demonstrated to play important roles in the infection and pathogenesis of many viruses. We previously found that porcine parvovirus (PPV) infection can induce autophagy in porcine placental trophoblast cells (PTCs), but its underlying mechanism has not yet been fully revealed. In this study, we showed that PPV infection inhibited the activation of mTORC1 and promoted the expression of Beclin 1 and LC3II in PTCs. Treatment with a mTOR activator inhibited the expression of Beclin 1 and LC3II, as well as autophagy formation, and reduced viral replication in PPV-infected PTCs. Furthermore, we found that inhibition of AMPK expression, but not the inhibition of PI3K/Akt, p53, or MAPK/ERK1/2 pathway activation, can significantly increase mTOR phosphorylation in PPV-infected PTCs. Then, we found that the regulation of mTOR phosphorylation by AMPK was mediated by Raptor. AMPK expression knockout inhibited the activation of Raptor, decreased the expression of Beclin 1 and LC3II, suppressed the formation of autophagosomes, and reduced viral replication during PPV infection. Together, our results showed that PPV infection induces autophagy to promote viral replication by inhibiting the activation of mTORC1 through activation of the AMPK/Raptor pathway. These findings provide information to understand the molecular mechanisms of PPV-induced autophagy.

**Supplementary Information:**

The online version contains supplementary material available at 10.1186/s13567-022-01048-7.

## Introduction

Porcine parvovirus (PPV) belongs to the genus *Protoparvovirus* in the family *Parvoviridae* and is a small nonenveloped virus with a single-stranded DNA genome [1, 2]. The PPV genome encodes three major nonstructural proteins (NS1, NS2, and NS3), three structural proteins (VP1, VP2, and VP3), and a late nonstructural protein (SAT) [3–6]. PPV is one of the major pathogens causing reproductive disorders in sows and is characterized by early embryonic death, foetal mummification, stillbirths, and infertility [7, 8]. Once pregnant sows are infected with PPV, the virus can cross the placental barrier and destroy the foetus [7]. Porcine placental trophoblasts (PTCs) are important components of the placental barrier and play a critical role in pregnancy maintenance. The dysfunction of PTCs is closely related to reproductive disorders [9–11]. Using PTCs as models to study the effects of PPV infection is an important way to study the mechanism of sow reproductive disorders caused by PPV infection.

Autophagy is an essential, conserved lysosomal degradation pathway in cells, but it is also involved in virus infection. However, depending on the viruses and host cell types, the regulatory mechanism of autophagy in various viral infections differs [12–14]. mTOR complex 1 (mTORC1) is a key regulator involved in multiple autophagy pathways and is composed of the core subunits mTOR, regulatory-associated protein of mTOR (Raptor), mammalian lethal with SEC13 protein 8 (mLST8), and two endogenous inhibitors of the complex, 40 kDa proline-rich Akt substrate (PRAS40) and DEP domain-containing mTOR-interacting protein (DEPTOR) [15–17]. mTORC1 activity is controlled by multiple signalling pathways, including the PI3K/Akt, adenosine 5′-monophosphate (AMP)-activated protein kinase (AMPK), MAPK/Erk1/2, and p53 pathways [18–20]. AMPK is a negative regulator of mTORC1, which can suppress mTORC1 by activating tuberous sclerosis complex 2 (TSC2) or phosphorylating the regulatory-associated protein of mTORC1 (Raptor) [21, 22]. Our recent study showed that PPV infection induces autophagy in PTCs [23]. Therefore, we were interested in determining whether mTORC1 is involved in PPV-induced autophagy and whether PPV-induced autophagy occurs via an AMPK-dependent mechanism. In this study, we used PPV-infected PTCs as models to explore the regulatory mechanism of autophagy induced by PPV. We found that PPV infection suppresses mTORC1 expression by phosphorylating Raptor with AMPK, which promotes PTC autophagy. Our study demonstrates that PPV infection activated AMPK-Raptor-mTOR-ULK1-Beclin1-dependent autophagy pathways.

## Materials and methods

### Cell culture and virus preparation

Placental trophoblast cells (PTCs) were isolated from healthy gilts and immortalized through reconstitution of telomerase in our previous study [9]. PTCs were cultured in Gibco Medium 199 (Cat No. 31100035, Gibco, USA), and PK-15 and HEK293T cells (ATCC, Manassas, VA, USA) were cultured in Dulbecco’s modified Eagle’s medium (DMEM) (Gibco, USA). All media were supplemented with 10% FBS (Cat No. 10099141, Gibco, USA), 100 U/mL penicillin, and 100 μg/mL streptomycin (Cat No. P1400, Solarbio, CHN) at 37 °C in an incubated with 5% CO_2_ atmosphere. PPV China-XY strain (MK993540) was propagated as previously described [23].

### Antibodies and inhibitors

Monoclonal antibodies against mTOR (Cat No. 29883), p-mTOR (Cat No. 5536), Beclin1 (Cat No. 3956), AMPK (Cat No. 5831), p-AMPK (Cat No. 50081), Raptor (Cat No. 2280), p-Raptor (Cat No. 2083) and LC3 (Cat No. 12741) were purchased from Cell Signaling Technology (CST, MA, USA). PFTα (a p53 inhibitor, Cat No.), Compound C (an AMPK inhibitor, Cat No. 171261), LY294002 (a PI3K/Akt inhibitor, Cat No. L9908), and PD98059 (an MAPK/ERK1/2 inhibitor, Cat No. P215) were purchased from Sigma–Aldrich (USA).

### Construction of the pCI-neo-Rheb recombinant vector

The *rheb* sequence (KF644436.1) was constructed from PTC cDNA and subcloned into a pCI-neo vector. The recombinant vector was digested and identified with restriction enzymes *Xho* I and *Xba* I. The pCI-neo-Rheb sequence was confirmed by sequencing analysis (Sangon Biotech, Shanghai, China).

### Western blotting

The cell pellets were lysed in RIPA with 1 mM PMSF and protease inhibitors (Sigma) to collect cellular protein. Similar amounts of protein from each extract were subjected to SDS–PAGE analysis and transferred to polyvinyl difluoride (PVDF) membranes (Millipore). After blocking for 1 h with blocking buffer (5% nonfat milk and 0.1% Tween-20 in PBS), the membranes were incubated with the following primary antibodies at 4 °C overnight: anti-p-mTOR, anti-mTOR, anti-Beclin1, anti-LC3, anti-p-AMPK, anti-AMPK, anti-p-Raptor, and anti-Raptor antibodies. HRP-conjugated anti-mouse IgG or anti-rabbit IgG (Boster Biological Technology Co. Ltd.) were used as secondary antibodies. ECL (Bio–Rad) was used for chemiluminescence detection according to the manufacturer’s instructions. ImageJ software was used to analyse and quantify the intensity of the protein bands.

### Specific inhibitor treatment

PTCs were pre-treated with solvent (DMSO), LY294002 (a PI3K/Akt inhibitor, 10 μM), Compound C (an AMPK inhibitor, 10 μM), PFTα (a p53 inhibitor, 10 μM), or PD98059 (an MAPK/ERK1/2 inhibitor, 20 μM) for 24 h, and cell viability was determined by MTT assay.

PTCs were pre-treated with solvent (DMSO), LY294002 (a PI3K/Akt inhibitor, 10 μM), Compound C (an AMPK inhibitor, 10 μM), PFTα (a p53 inhibitor, 10 μM), or PD98059 (an MAPK/ERK1/2 inhibitor, 20 μM) for 1 h, infected with PPV (MOI = 1), and subsequently cultured with M199 containing the corresponding inhibitor and 5% FBS.

### CRISPR–Cas9 mediated AMPK knockout in PTCs

According to the porcine AMPK sequence in NCBI, two pairs of gRNA sequences were designed (the sequence is shown in Figure 5A), and the synthesized gRNA sequence was phosphorylated and inserted into a lentiCRISPRv2 vector (#52961). Then, 293 T cells were transfected with the correct recombinant plasmid and packaging plasmid [pMD2]. G (#12259) and psPAX2 (#12260)], and the cell supernatant (containing recombinant lentivirus) was collected to obtain recombinant lentiviruses at 72 h. Then, the PTCs were infected with the recombinant lentiviruses. After 48 h, the cells were selected by puromycin at a concentration of 12 μg/mL. Positive cells were obtained after approximately 2 weeks and then subcloned into 96-well plates for single-clone growth and saved as cell stocks.

### Confocal microscopy detection of autophagy formation

For the detection of autophagosomes, GFP-LC3 plasmid-transfected PTC cells were mock-infected or infected with PPV for 12 h. Cells were washed with PBS, fixed with ice-cold 4% (wt/vol) paraformaldehyde for 20 min at room temperature, and then incubated with 0.1% Triton X-100 for 20 min, followed by staining with 4,6-diamidino-2-phenylindole (DAPI) nucleic acid stain. Then, fluorescence was observed under a laser scanning confocal microscope (Leica, TCS SP8).

### Quantitative PCR

Total RNA was extracted by TRIzol (Invitrogen, Carlsbad, CA, USA), and 500 ng of total RNA was reverse-transcribed into complementary DNA by using PrimeScript RT Master Mix (TaKaRa) according to the respective manufacturer’s protocol. The mRNA levels of *beclin1* and *lc3* were measured by quantitative PCR (q-PCR) with an Applied Biosystems QuantStudio 6&7 (Applied Biosystems, Grand Island, NY, USA) using SYBR Premix Ex Taq II DNA polymerase (TaKaRa), and GAPDH gene expression was used as the endogenous control. Specific primers used for *beclin1* and *lc3* were used for real-time PCR: *Sus scrofa beclin1* forward primer, 5′- CTGAAGAGTGTAG AAAACCAGATGC -3′, reverse primer 5′- CCAGCCTGAAGTTATTGATTGTG -3′, *S. scrofa lc3* forward primer, 5′- AACTAAGCTGTCTCTGCCCC -3′, reverse primer 5′- ACTGGGCCAGAATCCATCCA-3′. All samples were sequenced three times, and experiments were repeated three times.

### Statistical analysis

All experiments were performed at least three times, and the results are representative of three independent experiments. The data are presented as the means ± SEM (SD). Statistical significance was determined by ANOVA and Bonferroni post hoc test for multiple groups, while comparisons between 2 groups were analysed by unpaired Student’s *t* test. *P* < 0.05 was considered to be statistically significant.

## Results

### PPV infection suppresses mTOR phosphorylation

**Figure 1:**
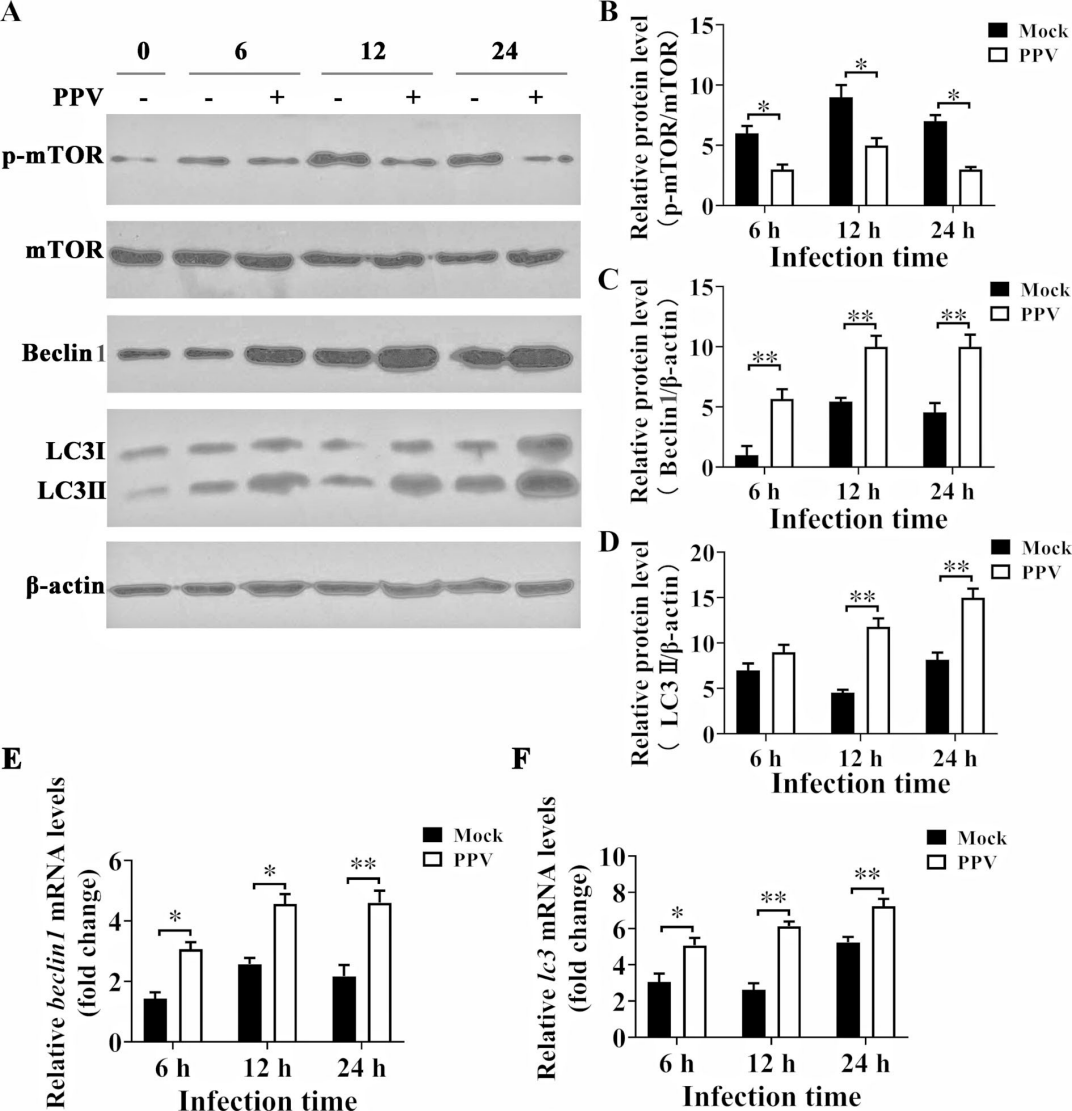
**PPV infection suppresses mTOR phosphorylation.** PTCs were infected with PPV at MOI = 1 or were mock infected for 0 h, 6 h, 12 h, and 24 h. **A** Cell lysates were collected individually for Western blotting analysis with anti-mTOR, anti-p-mTOR, anti-Beclin1, anti-LC3A/B and anti-β actin antibodies. **B**–**D** Ratios of p-mTOR to mTOR, as well as Beclin1 and LC3 II to β-actin, as reported in **A**, were calculated and analysed. **E**, **F** The mRNA levels of *beclin1* and *lc3* were detected by quantitative-polymerase chain reaction (q-PCR). The results are the mean ± SEM (SD) of three experiments, * *p* < 0.05; ** *p* < 0.01.

mTOR is a crucial regulator in the autophagy pathway [24]. To study the autophagy pathway regulated by PPV infection, we first explored whether mTOR is involved in PPV-induced autophagy. As the results showed, compared with those in the mock infection condition, the levels of p-mTOR in PTCs were all significantly decreased 6 h, 12 h, and 24 h post-PPV infection (Figures 1A and B). In contrast, PPV infection caused the protein levels of Beclin1 to begin to increase 6 hpi, and this increase persisted until 24 hpi in PTCs (Figures 1A and C), while the protein levels of LC3II began to increase 12 hpi (Figures 1A and D). Further, Q-PCR results showed that both *beclin1* and *lc3* mRNA levels were significantly upregulated in PTCs 6 h, 12 h, and 24 h post-PPV infection compared with those in the mock infection group (Figures 1E and F). These results suggest that mTOR is involved in the regulation of PPV-induced autophagy.

### Activation of mTOR inhibits PPV-induced autophagy and suppresses PPV replication

**Figure 2:**
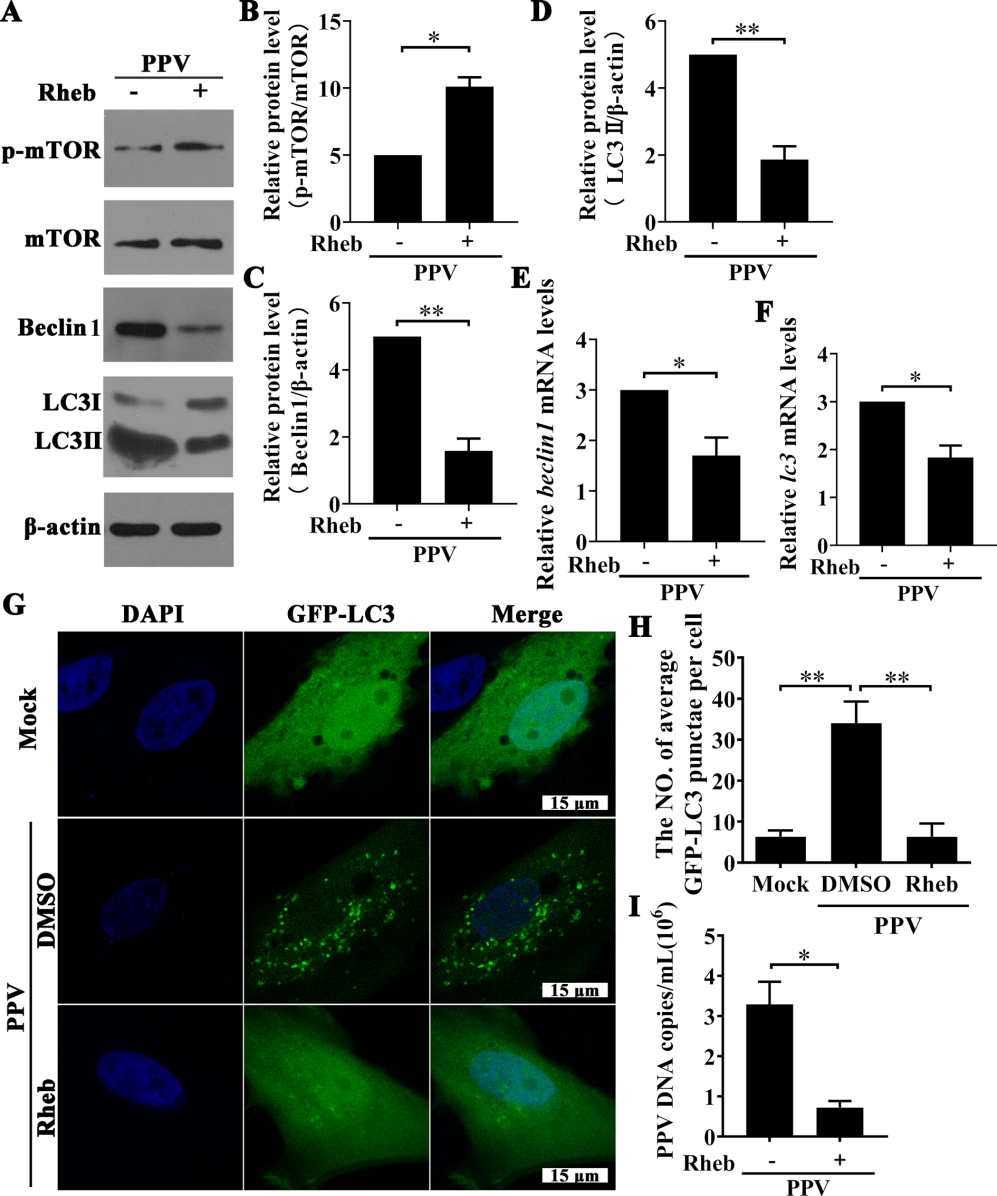
**Activation of mTOR inhibits PPV-induced autophagy and suppresses PPV replication**. PTCs were transfected with the mTOR activator Rheb plasmid or the same quality of vector and then infected with PPV for 12 h. **A** The levels of mTOR, p-mTOR, Beclin1 and LC3 II were examined by Western blotting. **B**–**D** Ratios of p-mTOR to mTOR, Beclin1 and LC3 II to β-actin as reported in **A** were calculated and analysed. **E**, **F** The mRNA levels of *beclin1* and *lc3* were detected by qPCR. **G**, **H** GFP-LC3-transfected PTCs were pre-treated with Rheb or DMSO. After infection with PPV or mock for 12 h, GFP-LC3 puncta formation in the cells was observed under laser scanning confocal microscopy (**G**). DAPI (blue) was used to stain nuclear DNA; scale bar: 15 μm. **H** The number of GFP-LC3 puncta in each cell was counted to calculate the average number of GFP-LC3 puncta per cell. At least 50 cells were counted in each group. **I** PTCs were infected with PPV after treatment or mock treatment with Rheb, and PPV DNA copies were then evaluated by Q-PCR 24 h post-infection. The results are the mean ± SEM (SD) of three experiments, * *p* < 0.05; ** *p* < 0.01.

Subsequently, to analyse whether inhibition of mTOR is an essential step in PPV-induced autophagy, PTCs were transfected with a eukaryotic recombinant plasmid expressing Rheb (Additional file 1), which positively regulates mTOR, before PPV infection. The results showed that the phosphorylation of mTOR increased notably in PPV-infected cells treated with Rheb compared with the PPV-infected cells without Rheb treatment (Figures 2A and B); however, Beclin1 and LC3 II expression was decreased significantly at the mRNA level (Figures 2E and F) and protein expression levels (Figures 2A–D) upon Rheb treatment in PPV-infected PTCs. Puncta formation of GFP-LC3-labelled vesicles is regarded as an indicator of autophagosome formation. As shown in Figures 2G and H, a large number of puncta were formed in GFP-LC3-labelled vesicles in PPV-infected PTCs, whereas puncta of GFP-LC3-labelled vesicles were rarely observed upon Rheb treatment of PPV-infected PTCs, indicating that inhibition of mTOR was an essential step in PPV-induced autophagy. Furthermore, we found that Rheb treatment not only inhibited autophagosome formation but also inhibited PPV replication in PTCs (Figure 2I). Taken together, these data indicate that PPV infection induces autophagy and promotes PPV replication by inhibiting mTOR signalling.

### The AMPK pathway acts as a key regulator to suppress mTOR phosphorylation during PPV infection

**Figure 3:**
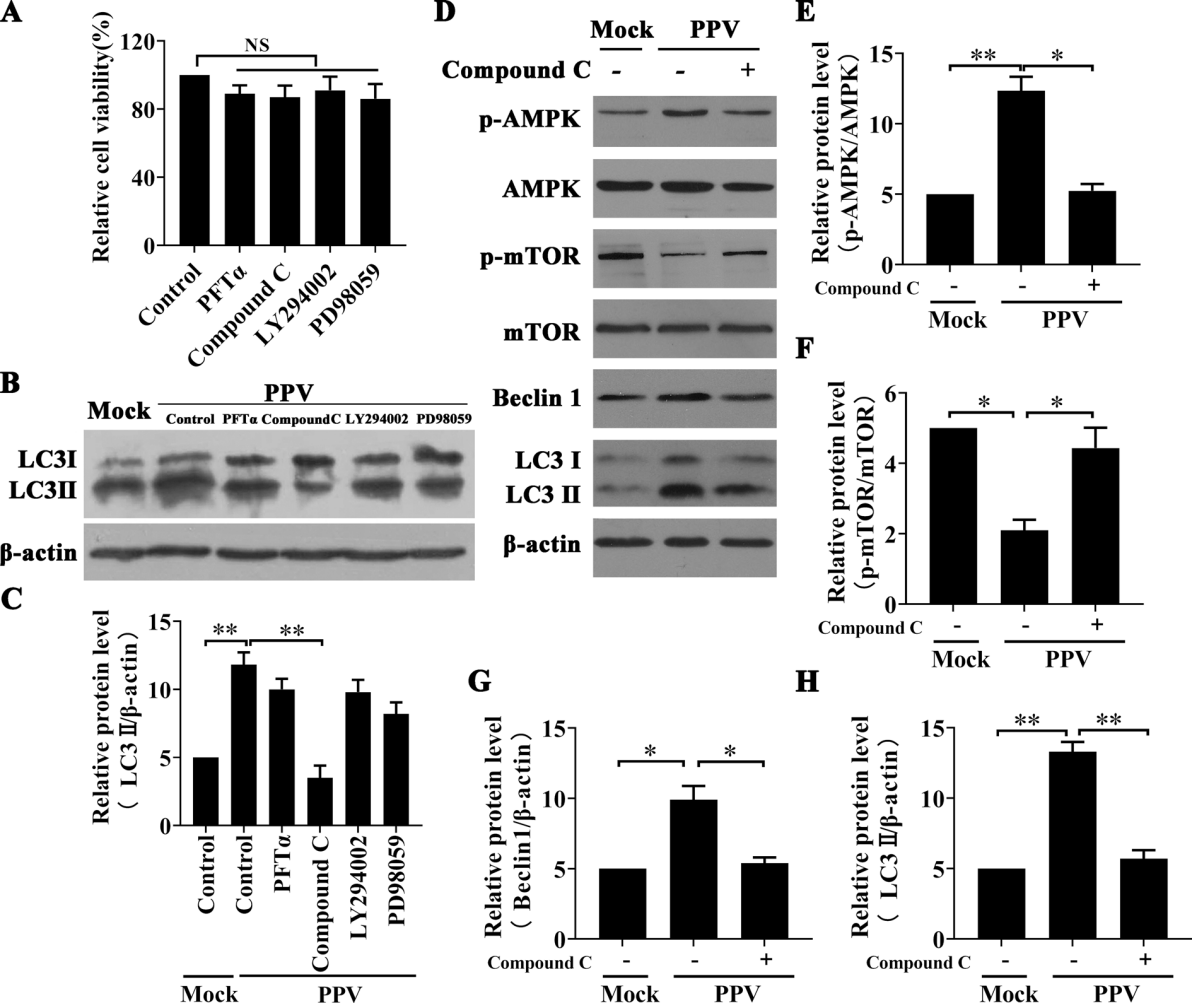
**The AMPK pathway acts as a key regulator for suppressing mTOR phosphorylation during PPV infection**. **A**, **B** PTCs were pre-treated with solvent (DMSO), LY294002 (a PI3K/Akt inhibitor, 10 μM), PFTα (a p53 inhibitor, 10 μM), Compound C (an AMPK inhibitor, 10 μM), PFTα (a p53 inhibitor, 10 μM), or PD98059 (a MAPK/ERK1/2 inhibitor, 20 μM), and cell viability was determined by MTT assay (**A**). Then, PTCs were infected with PPV (MOI = 1) for 12 h after being pre-treated with the above inhibitors. The level of LC3 II was evaluated by Western blotting (**B**), and the ratio of LC3 II/β-actin was calculated and analysed (**C**). (D-H) PTCs were pre-treated with Compound C (10 μM) or the same volume of DMSO and then infected with PPV (MOI = 1) or mock for 12 h. The levels of the indicated cellular proteins were evaluated by Western blotting (**D**), and the ratios of p-AMPK to AMPK, p-mTOR to mTOR, and Beclin1 and LC3 II to β-actin as reported in **D** were calculated and analysed (E–H). The results are presented as the mean ± SEM (SD) of three experiments, * *p* < 0.05; ** *p* < 0.01.

Four classical pathways are involved in regulating autophagy through the regulation of mTOR expression [17, 24, 25]. To explore the upstream regulator of mTOR expression during PPV infection, PTCs were pre-treated with LY294002 (PI3K/Akt inhibitor, 10 μM), Compound C (AMPK inhibitor, 10 μM), PFTα (p53 inhibitor, 10 μM), or PD98059 (MAPK/ERK1/2 inhibitor, 20 μM) and then infected with PPV for 12 h. The results showed that the above inhibitors did not induce toxic effects in cells when administered at the indicated concentration (Figure 3A). The inhibition of AMPK expression with Compound C significantly decreased the level of LC3 II, whereas the inhibitors of the PI3K/Akt, p53, and MAPK/Erk1/2 signalling pathways exerted no significant effect (*p* > 0.05) on the level of LC3 II in the PPV-infected cells (Figures 3B and C). Furthermore, PPV infection increased the ratio of phosphorylated AMPK relative to the number of uninfected cells, and inhibition of AMPK phosphorylation with Compound C re-established mTOR phosphorylation and downregulated Beclin1 and LC3 II expression in PPV-infected PTCs (Figures 3D–H), suggesting that PPV infection activates AMPK phosphorylation to promote autophagy by suppressing mTOR phosphorylation in PTCs.

### PPV infection induces Raptor phosphorylation by activating the AMPK signalling pathway

**Figure 4:**
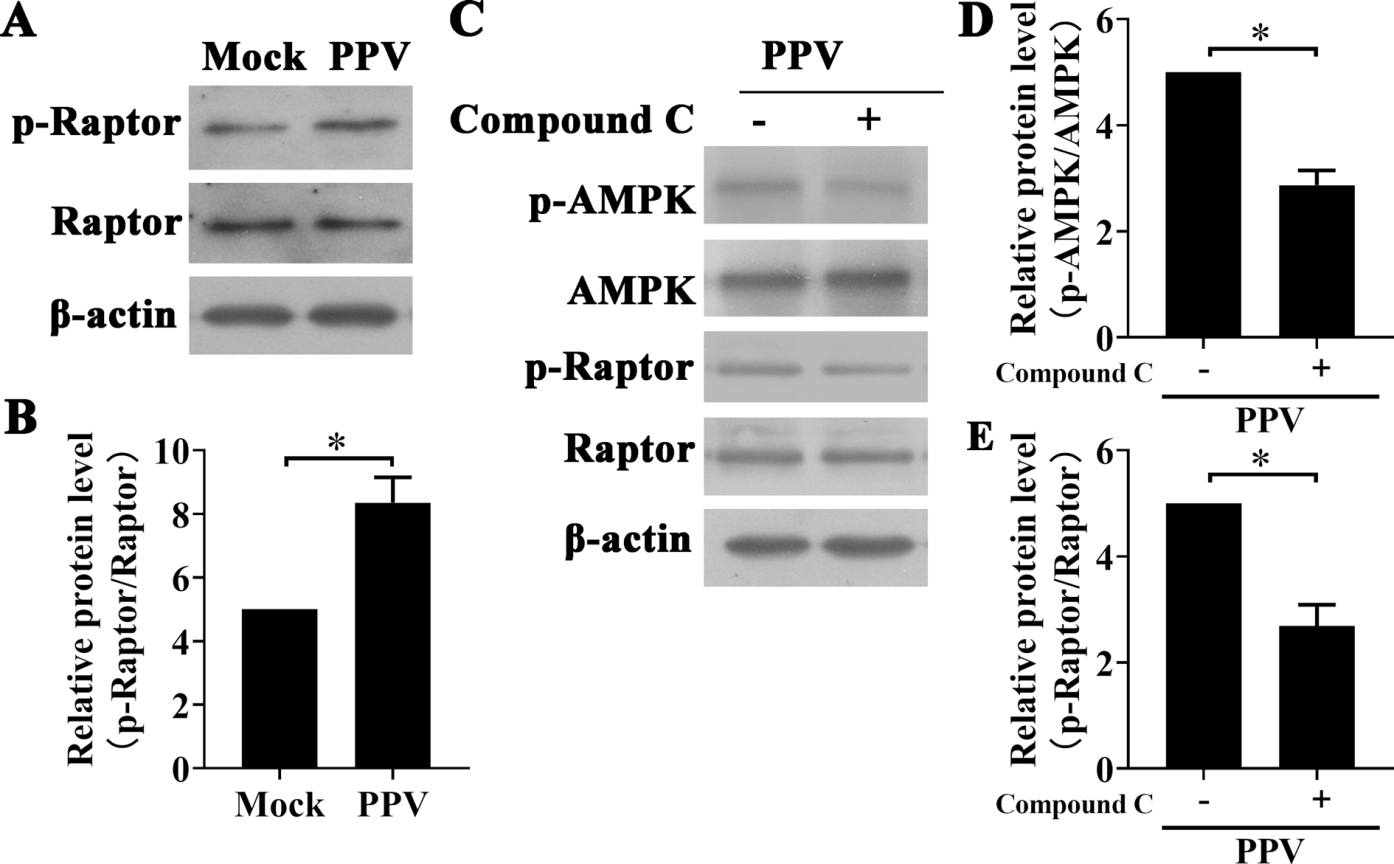
**PPV infection induces Raptor phosphorylation by activating AMPK phosphorylation**. **A**, **B** PTCs were infected with PPV or mock for 12 h. Raptor and p-Raptor were evaluated via Western blotting (**A**), and the ratio of p-Raptor/Raptor in cells was calculated and analysed (**B**). **C**–**E** PTCs were pre-treated with Compound C (10 μM) and then infected with PPV (MOI = 1) for 12 h. The levels of the cellular proteins indicated were evaluated by Western blotting (**C**), and the ratios of p-AMPK to AMPK and p-Raptor to Raptor as reported in **D**, **E** were calculated and analysed. The results are presented as the mean ± SD of three experiments, * *p* < 0.05; ** *p* < 0.01.

AMPK is a negative regulator of mTORC1, which indicates that it can suppress mTORC1 expression by activating tuberous sclerosis complex 2 (TSC2) or phosphorylating the regulatory-associated protein of mTORC1 (Raptor) [21]. In our study, we found that PPV infection decreased Raptor phosphorylation (Figures 4A and B). Inhibition of AMPK phosphorylation with Compound C markedly decreased the level of Raptor phosphorylation and recovered mTOR phosphorylation in PPV-infected PTCs (Figures 4C–E), indicating that PPV infection induces Raptor phosphorylation by activating AMPK phosphorylation. These data suggest that AMPK suppresses mTORC1 by phosphorylating Raptor in PPV-infected PTCs.

### AMPK deficiency reduced autophagy and viral replication in PPV-infected PTCs

**Figure 5:**
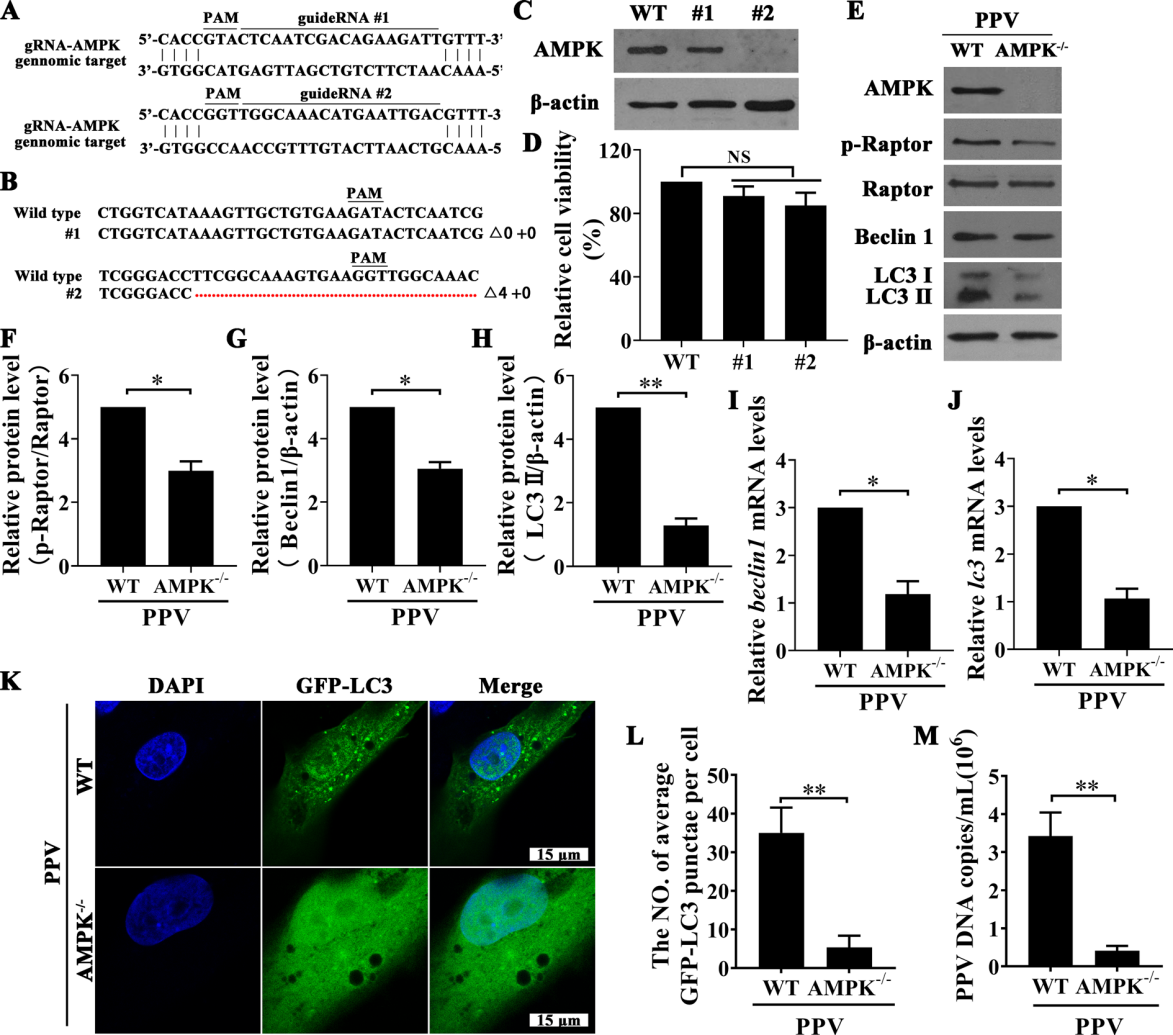
**Knockout of AMPK expression reduced autophagy and PPV DNA copies in PPV-infected PTCs**. **A** Schematic diagram of gRNA targeting sites in the AMPK genomic region. **B**–**D** PTCs were treated with a CRISPR–Cas9 system targeting the AMPK locus. Two single-cell clones (#1 and #2) were derived from cells infected with lentiviral pseudotypes expressing gRNAs 1 and 2, respectively. Then, the target site was sequenced/; + means insertion, △ means deletion, red dotted line presents deleted fragments (**B**) and AMPK expression in PTCs was determined (**C**), and cell viability was determined by MTT assay (**D**). (E–H) Wild-type (WT) PTCs and AMPK^−/−^ PTCs were infected with PPV, and the proteins indicated were evaluated by Western blotting (**E**), and the ratios of the proteins indicated were calculated and analysed (**F**–**H**). **I**, **J** The mRNA levels of *beclin1* and *lc3* were detected by qPCR. **K**, **L** WT PTCs and AMPK^−/−^ PTCs were transfected with a GFP-LC3 plasmid and then infected with PPV for 12 h. GFP-LC3 puncta formation was then observed under laser scanning confocal microscopy (**K**). DAPI (blue) was used to stain nuclear DNA; scale bar: 15 μm. The number of GFP-LC3 puncta in each cell was counted, and at least 50 cells were counted in each group. Next, the average number of GFP-LC3 puncta per cell was calculated (**L**). **M** WT PTCs and AMPK^−/−^ PTCs were each infected by PPV, and PPV DNA copies were evaluated by Q-PCR 24 h post-infection. The results are presented as the mean ± SEM (SD) of three experiments, * *p* < 0.05; ** *p* < 0.01.

To further elucidate the role played by AMPK in promoting PPV-induced autophagy, we generated AMPK^−/−^ stable cell lines using the CRISPR/Cas9 system (Figures 5A–D). The level of Raptor phosphorylation, as well as the levels of Beclin1 and LC3 II expression, were significantly decreased in PPV-infected AMPK^−/−^ cells compared to those in PPV-infected wild-type cells (Figures 5E–J). Knockout of AMPK expression also led to reduced puncta formation in GFP-LC3-labelled vesicles and fewer PPV DNA copies in PPV-infected PTCs (Figures 5K–M), indicating that PPV infection induces autophagy to promote viral replication by activating the AMPK/Raptor pathway.

## Discussion

Autophagy is a multistep precisely regulatory process involving multiple signalling molecules [26]. mTOR is a key regulatory hub in autophagy. Activating mTOR (PI3K-I/AKT or MAPK/Erk1/2 signal transduction) can inhibit autophagy, and inhibiting mTOR (p53 or AMPK signal transduction) can promote cell autophagy. The Beclin-1 complex (Beclin-1-Vps15-Vps34) is a downstream target of mTOR and plays a central role in the initiation of autophagy [27]. The expression of Beclin-1 is regulated at the level of transcription, translation, and post-translational modification. Studies have shown that upregulating the expression of Beclin-1 in mammalian cells can induce autophagy [28]. This study found that PPV infection with PTCs leads to a decrease in the p-mTOR level and an increase in the Beclin-1 expression level, which means that PPV inhibits the phosphorylation of mTOR, thereby enhancing autophagy. Rheb pre-treatment partially activates the mTOR pathway, reduces the expression level of Beclin-1, and inhibits the promotion of autophagy by PPV. These results indicate that PPV can induce autophagy by upregulating the expression of Beclin-1 in cells by inhibiting mTOR phosphorylation.

**Figure 6:**
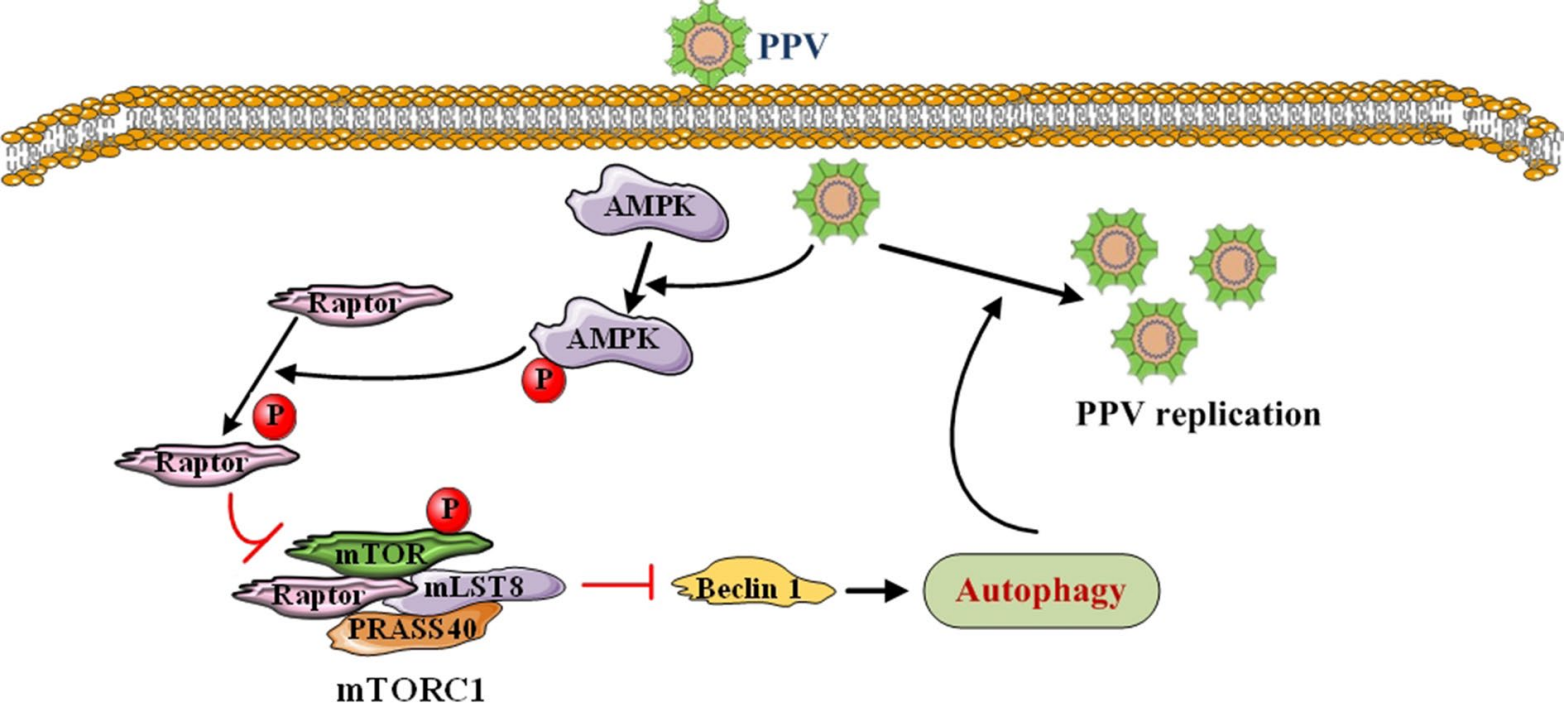
**The model of autophagy induced by PPV infection**. PPV infection inhibits mTORC1 by activating the AMPK/Raptor pathway, promotes the expression of Beclin1 and LC3 II, and induces autophagy to promote viral replication.

AMPK is a key sensor of cellular energy status and a major regulator of metabolism, and it plays a key role in energy homeostasis [29]. In recent years, AMPK has been shown to be closely related to autophagy. AMPK regulates various aspects of the autophagy mechanism, and phosphorylation of AMPK reduces the inhibitory phosphorylation of ULK1 by reducing mTOR activity to activate autophagy [30]. This study showed that PPV infection of PTCs increased the level of p-AMPK, which meant that PPV promoted the phosphorylation of AMPK, thereby enhancing autophagy. After Compound C pre-treatment, the AMPK pathway was partially blocked, and the promotion of autophagy induced by PPV was inhibited. This result also indicates that PPV regulates autophagy by activating the AMPK signalling pathway. After AMPK is activated, it phosphorylates TSC2 and Raptor, which leads to the downregulation of mTOR complex 1 (mTORC1) expression and thus blocks the ability of the mTORC1 kinase complex to phosphorylate its substrates. The mTORC1 complex is composed of mTOR, Raptor, mLST8, DEPTOR, and PRAS40. As a binding protein of mTOR, Raptor regulates mTOR activity by binding to the downstream substrates of mTOR, 4EBP1, and P70S6K. In this study, PPV was used to infect PTCs, and the p-Raptor level in the PTCs was increased. Raptor inhibitors inhibited the decrease in mTOR phosphorylation, the increase in Beclin1 expression, and the increase in LC3II expression caused by PPV infection, but there was no significant effect on AMPK activity. The results also showed that PPV infection can activate AMPK and inhibit the mTORC1 signalling pathway through Raptor, activate autophagy, and induce high expression of Beclin-1 and LC3 II (Figure 6).

## Supplementary Information

**Additional file 1**: **Construction and identification of the recombination plasmid pCI-neo-Rheb.** (A)rhebwas amplifiedthrough PCR assay, and then the rhebfragment was inserted intoapCI-neovector.(B) The recombinant plasmid pCI-neo-Rheb wassubjected to restriction enzyme digestion identification. M1: DL2000 plus DNA Marker; 1: PCR of rheb; M1: DL10000 plus DNA Marker; 2: digested detection of pCI-neo-Rheb; The arrow points to the destination band.

## Data Availability

All data generated or analysed during this study are included in this published article.
