# Supplementary figures and images for: Porcine parvovirus triggers autophagy through the AMPK/Raptor/mTOR pathway to promote viral replication in porcine placental trophoblasts
Source: Vet Res. 2022 May 3;53:33. doi: 10.1186/s13567-022-01048-7 (PMC9066968; doi:10.1186/s13567-022-01048-7)

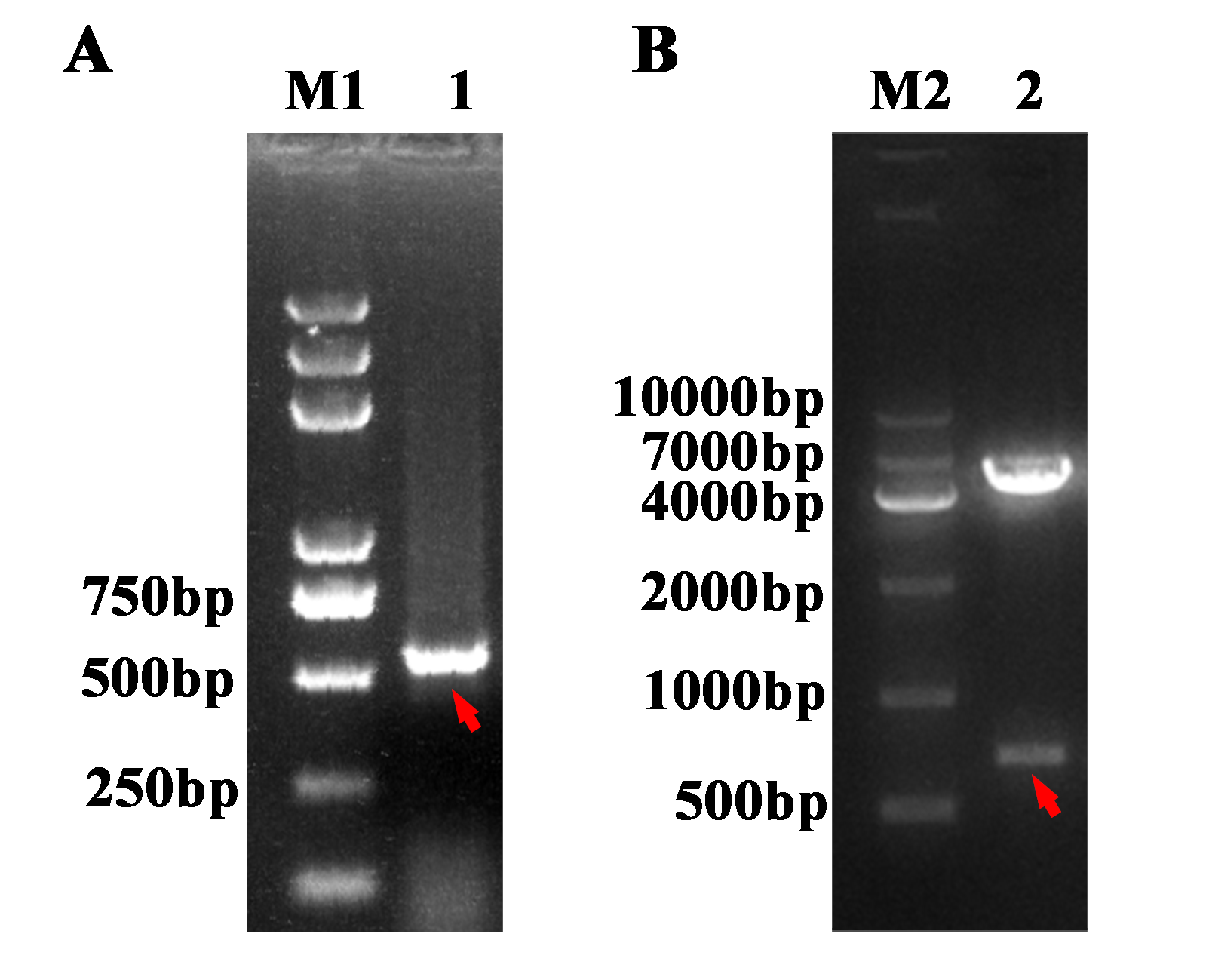

Supplement: Supplementary file 1 — Additional file 1: Construction and identification of the recombination plasmid pCI-neo-Rheb. (A)rhebwas amplifiedthrough PCR assay, and then the rhebfragment was inserted intoapCI-neovector.(B) The recombinant plasmid pCI-neo-Rheb wassubjected to restriction enzyme digestion identification. M1: DL2000 plus DNA Marker; 1: PCR of rheb; M1: DL10000 plus DNA Marker; 2: digested detection of pCI-neo-Rheb; The arrow points to the destination band. [file 13567_2022_1048_MOESM1_ESM.doc]
